# Author Correction: Seasonal advance of intense tropical cyclones in a warming climate

**DOI:** 10.1038/s41586-025-08691-y

**Published:** 2025-02-19

**Authors:** Kaiyue Shan, Yanluan Lin, Pao-Shin Chu, Xiping Yu, Fengfei Song

**Affiliations:** 1 State Key Laboratory of Hydroscience and Engineering, Department of Hydraulic Engineering, Tsinghua University, Beijing, China; 2 Department of Earth System Science, Ministry of Education Key Laboratory for Earth System Modeling, Institute for Global Change Studies, Tsinghua University, Beijing, China; 3 Department of Atmospheric Sciences, School of Ocean and Earth Science and Technology, University of Hawai’i at Mānoa, Honolulu, HI USA; 4 Department of Ocean Science and Engineering, Southern University of Science and Technology, Shenzhen, China; 5 Frontier Science Center for Deep Ocean Multispheres and Earth System and Physical Oceanography Laboratory, Ocean University of China, Qingdao, China; 6 Laoshan Laboratory, Qingdao, China

**Keywords:** Climate-change impacts, Natural hazards

Correction to: *Nature* 10.1038/s41586-023-06544-0 Published online 27 September 2023

In our trend analysis of intense tropical cyclone (TC) occurrences, we calculated the number of days between the median value of intense TC occurrence date and start date of that TC season, which requires the precise number of days in each month. In the calculation, we converted the TC occurrence dates for the Southern Hemisphere (SH) by adding six months, because we define the SH TC season as July 1 of the previous year to June 30 of the current year. However, we mistakenly used the number of days for each month as if starting from January, instead of from July. Additionally, we did not account for the effect of leap years on February’s day count. Consequently, the estimated number of days between the median intense TC occurrence date and start date of the TC season was underestimated. Furthermore, during the preprocessing of the best-track data, four intense TCs were mistakenly omitted. We have since recalculated the trend of intense TC occurrences and adjusted the results in the article accordingly. We thank Jimin Liu (Lanzhou University, National University of Defense Technology, Guangzhou Institute of Tropical and Marine Meteorology), Jeremy Cheuk-Hin Leung (Guangzhou Institute of Tropical and Marine Meteorology), Wenshou Tian (Lanzhou University), Hong Huang (National University of Defense Technology), Daosheng Xu (Guangzhou Institute of Tropical and Marine Meteorology), Weijing Li (Guangzhou Institute of Tropical and Marine Meteorology), Weihong Qian (Guangzhou Institute of Tropical and Marine Meteorology, Peking University), Banglin Zhang (National University of Defense Technology, Lanzhou University) for alerting us to these errors. The issues mentioned above slightly affect the estimated value of the earlier-shifting trend of intense TCs but do not affect our conclusions.

With the modifications applied, the rate of the earlier-shifting trend of intense TCs in the SH is now 3.0 days per decade, compared to the previous estimate of 3.2 days per decade based on the ADT-HURSAT dataset, while there is no change in the Northern Hemisphere (NH). These issues also influence the estimated values of the earlier-shifting trend derived from the best-track data, as shown in the updated Table 1. We emphasize that this correction does not alter the main conclusions outlined in the published paper. Furthermore, a simple test assuming each month has 30 days demonstrated a robust seasonal advance of intense TCs.

When building on this research, we prioritized the ADT-HURSAT dataset and used the best-track data as supporting evidence, given the significant spatial and temporal heterogeneity in the best-track dataset. Due to this heterogeneity, we cannot ensure the reliability of the higher-intensity data from the best-track dataset when using it for trend analysis. It is observed that the number and proportion of intense TC cases tend to be overestimated in the best-track dataset when using the threshold value of 110 kt for intense TCs, compared to the ADT-HURSAT dataset. To address this issue and ensure comparability, we adopted a slightly higher threshold value of 115 kt for intense TCs in the best-track dataset. This adjustment helps align the proportion of intense TCs between the datasets. Additionally, part of the 2021 TC best-track data was not in its final form when we accessed the dataset on May 29, 2022. As a result, the median value for the 2021 TC season should be excluded. The trend exhibits some fluctuations but remains statistically significant when TCs exceed the intensity of intense TCs. This is primarily attributed to the smaller sample size at higher intensities, which may result in greater variability in trend estimation. These explanations accurately reflect the analyses performed.

The recalculation affects data reported in Fig. 2, Table 1 and Extended Data Fig. 2, which are all updated in the HTML and PDF versions of the article. For comparison, original version of the figures and table are available in the Supplementary information accompanying this amendment.

## Supplementary information

Supplementary Information Original and revised Fig. 2, Table 1, Extended Data Fig. 2.

