# Supplementary material for: Author Correction: Seasonal advance of intense tropical cyclones in a warming climate
Source: Nature. 2025 Feb 19;639(8053):E4. doi: 10.1038/s41586-025-08691-y (PMC11882436; doi:10.1038/s41586-025-08691-y)

nature portfolio

https://doi.org/10.1038/s41586-025-08691-y

Supplementary information

Author Correction: Seasonal advance of intense tropical cyclones in a warming climate

In the format provided by the authors and unedited

Supplementary information

Original Figure 2.

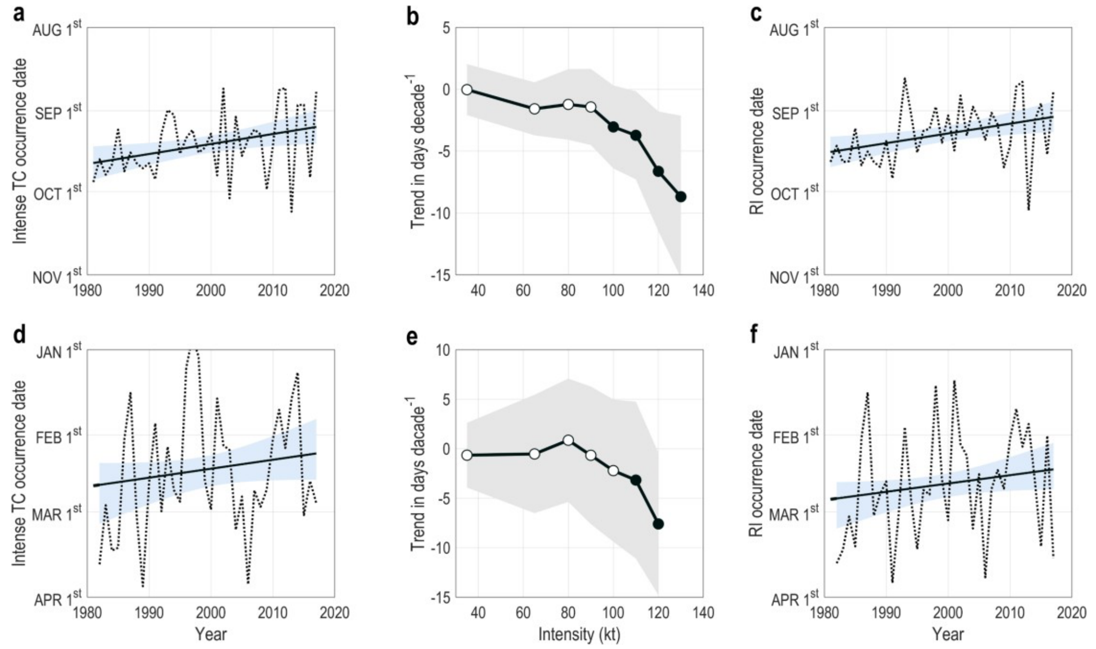

Updated Figure 2.

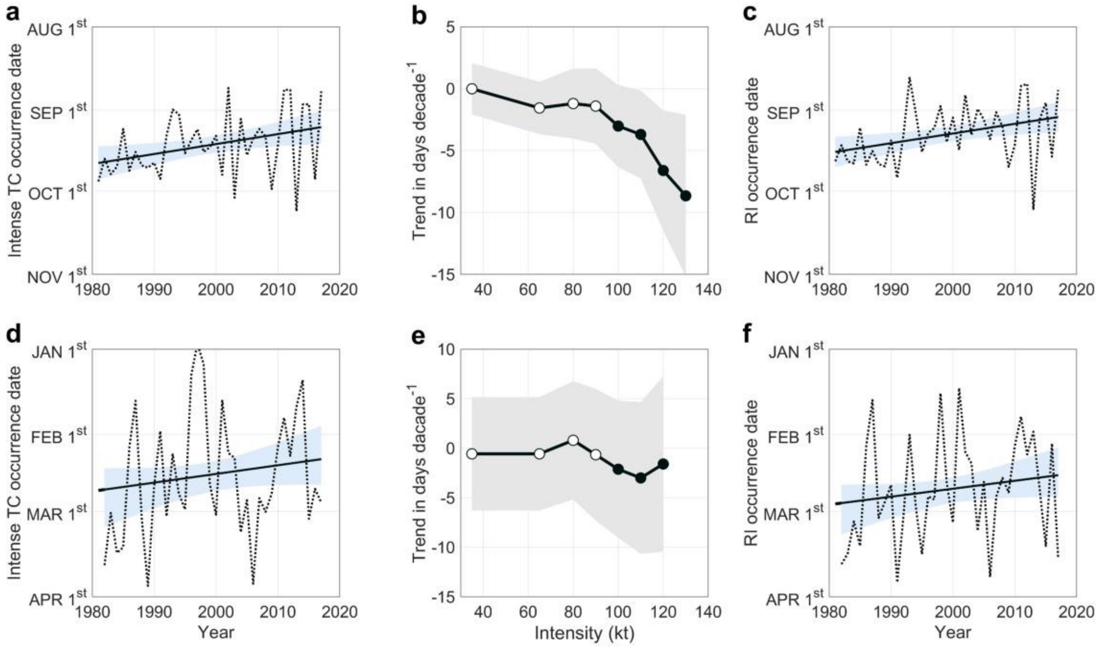

Original Table 1.

		Trends (day decade^{-1})	
		Median	μ
ADT-HURSAT	NH	-3.7* ± 3.6	-3.4* ± 2.9
	SH	-3.2* ± 7.9	-3.1* ± 3.4
Best-track	NH	-2.3* ± 2.1	-2.7* ± 2.6
	SH	-4.1* ± 3.9	-1.8* ± 3.5

Updated Table 1.

		Trends (day decade^{-1})	
		Median	μ
ADT-HURSAT	NH	-3.7* ± 3.5	-3.4* ± 2.9
	SH	-3.0* ± 7.7	-3.1* ± 3.4
Best-track	NH	-2.4* ± 2.2	-2.7* ± 2.6
	SH	-3.8* ± 4.2	-1.8* ± 3.5

Original Extended Data Figure 2.

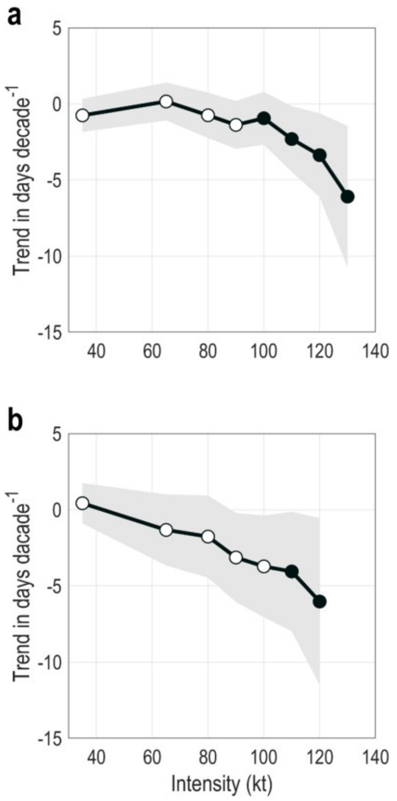

Updated Extended Data Figure 2.

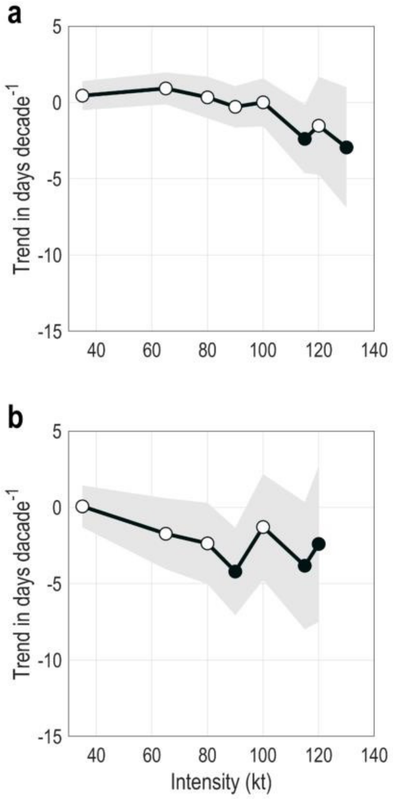

Supplement: Supplementary file 1 — Original and revised Fig. 2, Table 1, Extended Data Fig. 2. [file 41586_2025_8691_MOESM1_ESM.pdf]
